# Strong association of anemia in people with diabetic foot ulcers (DFUs): Study from a specialist foot care center

**DOI:** 10.12669/pjms.35.5.1421

**Published:** 2019

**Authors:** Abdul Majid Shareef, Muhammad Yakoob Ahmedani, Nazish Waris

**Affiliations:** 1 Dr. Abdul Majid Shareef, MBBS. Podiatric Surgeon, Department of Foot, Baqai Institute of Diabetology and Endocrinology (BIDE), Baqai Medical University (BMU), Karachi-Pakistan; 2 Prof. Muhammad Yakoob Ahmedani, FCPS. Professor of Medicine (BMU), Department of Medicine, Baqai Institute of Diabetology and Endocrinology (BIDE), Baqai Medical University (BMU), Karachi-Pakistan; 3 Dr. Nazish Waris, Ph.D. Research Officer, Research Department, Baqai Institute of Diabetology and Endocrinology (BIDE), Baqai Medical University (BMU), Karachi-Pakistan

**Keywords:** Diabetic foot ulcer, Type 2 diabetes, Anaemia

## Abstract

**Background & Objective:**

Anemia is common finding in people with diabetes and diabetic foot ulcers. Therefore, our objective was to observe and compare the association of anemia in people with diabetic foot ulcers (DFUs) with age and sex matched people without DFUs.

**Method:**

This prospective case control study was undertaken at a multidisciplinary diabetic foot clinic of Baqai Institute of Diabetology and Endocrinology (BIDE) between October 2014 and October 2015. Participants were categorized into two groups; Case group (people with DFUs) and Control group (people without DFUs). Baseline demographic characteristics, biochemical and hematological parameters were recorded. University of Texas (UT) classification system was used to grade and stage DFU in case group. Subjects with other apparent causes of anaemia were excluded. Age and sex matched controls were taken from diabetic clinic presented during same time period. Data was analyzed by using statistical package for social science (SPSS) version 20.

**Results:**

Total of 161 participants in case group were compared with similar number of age and sex matched participants of control group. Most of the participants were males 119(73.9%). Age and sex matched case and control groups were comparable except for duration of diabetes, BMI and HDL levels. Overall, 85.67% case group (males 64.56%); females 21.11%) and 35.3% control group (males 22.9%; females12.4%) have anemia. Mean Hb level was 10.49g/dl in case group and 13.39g/dl in control group. Significant differences were also noted in other blood parameters.

**Conclusion:**

Our study concludes that anaemia is strongly associated with DFU disease. Anaemia should be considered and treated as co-morbidity while managing patients with foot ulcers.

## INTRODUCTION

Diabetic foot is a common, serious limb-threatening complication and it is considered as a major cause of morbidity and hospitalization in patients with diabetes. Nearly, 4% to 10% of people with diabetes develop foot ulcer.^1^ The lower extremity amputation (LEA) rate following foot ulceration has been reported to be 8–21%.^2^,^3^ Diabetic foot disease carries huge economic burden and significant psychosocial impact.^4^,^5^

Peripheral vascular disease, severity of neuropathy, structural foot deformity, concomitant infection, high plantar pressure, poor glycemic control, duration of diabetes, male gender and presence of other micro and macrovascular complications are common risk factors for foot ulceration.^6^ Outcomes of DFUs are closely related to the severity of disease. Among other common factors anemia is also considered one of the major predictor of outcome of DFU.^7^ In people with diabetes and DFU, pathogenesis of anemia has a number of adverse consequences and they can be varied. Anaemia in diabetes is frequently unrecognized and has shown that it is twice as common in people with diabetes as compared to people without diabetes.^8^ Similarly, morbidity and mortality rates in people with DFU along with anemia has been shown to increase incidence as compared to people without anemia.^9^

### Key points

- Anemia is considered as one of the major predictor of outcome of DFU.
- Association of anemia in the people with DFUs with age and sex matched people without DFUs were compared in this study.
- Anaemia should be screened and considered as co-morbidity while managing patients with foot ulcers.

It was reported in Bates M et al. study that anaemia in people with DFU delay or results in deterioration of wound healing.^10^ Frequency of anemia in people with type 2 diabetes was observed 63% in Sharif A et al study and in people with DFU it is 59.04% in Wright JA et al. study, while 89.6% anemic people with DFU underwent amputations in Costa RH et al. study.^8^,^11^,^12^ Limited studies have been done to observe the association of anaemia with DFU.^13^ To the best of our knowledge, none of the studies from Pakistan reported it. Moreover, there is no comparative data to suggest the presence or absence of anaemia in age and sex matched type 2 diabetes individuals without foot ulcers. In the present study, we aimed to observe and compare the association of anemia in- the people with DFUs with age and sex matched people without DFUs.

## METHODS

This prospective case control study was conducted at Baqai Institute of Diabetology and Endocrinology (BIDE), Baqai Medical University (BMU), a tertiary care unit of Karachi- Pakistan. People with diabetes attending outpatient department (OPD) of foot clinic or admitted in ward with diagnosis of DFU between October 2014 to October 2015 were recruited. Age and sex matched controls were recruited from diabetic clinic during same time period after obtaining informed consent. Both cases and control groups taking no treatment or treatment of oral hypoglycemic agents (OHA) or insulin or both were included. Ethical approval was obtained by Institutional Review Board (IRB) of BIDE. People without diabetes or having Type-1 diabetes, all pregnant females were excluded from this study. People having hematological disorders and already taking iron or folic acid or B12 supplements were excluded from this study. Patients with DFUs having wounds secondary to major trauma like road traffic accident or having other causes of anaemia including chronic liver disease, malabsorption, inadequate meat intake, strict vegetarian, were excluded from this study. Patients with DFU having chronic renal failure (CRF) were also excluded. Data was collected on questionnaire by associate physician.

Study participants were categorized into two groups; Case group (people with DFU) and Control group (people with diabetes without DFU). Convenient sampling technique was adopted. Baseline demographic parameters including gender, age, duration of diabetes, family history of diabetes, body mass index (BMI) and systolic and diastolic blood pressure were noted. The blood samples for CBC were taken at the time of the patient’s arrival in OPD or at admission in the ward before any debridement was done as it may cause loss of blood and anemia in the patients with DFU. Biochemical and hematological parameters including glycated hemoglobin (HbA1c), lipid profile, serum creatinine and complete blood count (CBC) were recorded.

Anaemia was defined by World Health Organization (WHO) as haemoglobin (Hb) levels <13g/dL for males and <12 g/dL for females.^14^ Comorbidities, detailed information including family, diet, personal and drug history was taken. Anemia was categorized by red cell size as microcytic (MCV <80 fl), normocytic (normal MCV 80-100 fl) or macrocytic (MCV >100 fl).^15^ People having microcytic anemia were further assessed for serum ferritin and serum iron levels.^16^,^17^ Foot ulcer in case group was evaluated according to University of Texas (UT) diabetic wound classification from grade 0 to Grade-III with four stages in each grade.^18^ Patient with epithelialized wound or with feet at risk as grade 0, superficial wound as Grade-I, deep wound involving tendons but not involving bone as Grade-II, wounds that involves bones, localized and generalized gangrene as Grade-III. No infection and ischemia (stage A), presence of infection (stage B), presence of ischemia (stage C) and presence of both infection and ischemia (stage D).

Complete blood count (CBC) was done on the automated hematology analyzer (MEK-6450 Celltac α Nihon Kohden). Glycemic control was assessed by HbA1c using HPLC method on BIO RAD D-10. To determine triglycerides, GOD- PAP method on selectra Pro Sa fully automated analyzer was used. Serum total cholesterol was analyzed by Chod-Pap method on selectra pro s; a fully automated analyzer. Homogeneous enzymatic colorimetric method was used for high density lipoprotein (HDL)–cholesterol and Direct method used for low density lipoprotein (LDL)-cholesterol measurement.

Height was measured to the nearest of 0.1cm, while subject standing in erect posture and weight was measured with portable weighing scale nearest of 0.1 kilogram (kg). Body mass index (BMI) was measured as the ratio of weight (kg) to height squared (m^2^). As per Asian guideline, people having BMI ≥ 25 (kg/m^2^) were labeled obese.^19^ Blood pressure of the participants was monitored by mercury sphygmomanometer in a sitting position by using standard method. Hypertension was defined as blood pressure ≥130/85mmHg.^20^

### Data analysis

Data was analyzed by using Special Package for Social Sciences (SPSS) version 20.0. Frequencies and percentages were computed for categorical variables. Mean ±Standard Deviation were computed for numerical variables. Chi square test was performed at 95% Confidence Interval and used to compare relative frequencies of categorical variables. Student t-test was used to compare Mean ± Standard Deviation of numerical variables. Level of significance was taken as p<0.05.

## RESULTS

Total of 208 DFUs cases were recruited in case group. Of these cases 47 were excluded due to presence of other apparent causes of anaemia in them. Remaining 161 cases in case group were compared with similar number of age and sex matched people with type 2 diabetes without DFU.

Majority of the participants were malesi.e.73.9%. Total of 3.7% participants were found between 30-40 years, 15.5% between 40-50 years and 80.7% in ≥ 50 years of age. Age and sex matched control groups were comparable. No significant differences were observed for systolic and diastolic blood pressure, total cholesterol, T.G, LDL, serum creatinine and HbA1c between the both two groups. (Table-I)

**Table I:** Basic Demographic and Biochemical characteristics of case and control group.

Variables	Case	Control	P-value
N	161	161	-
***Age (in years)***			
30 – 40	6(3.7%)	6(3.7%)	0.999
40 -50	25(15.5%)	25(15.5%)	
≥ 50	130(80.7%)	130(80.7%)	
***Gender***			
Male	119(73.9%)	119(73.9%)	0.999
Female	42(26.1%)	42(26.1%)	
***Duration of diabetes (in years)***			
<5	27(16.8%)	34(21.1%)	0.001
5 – 10	28(17.4%)	50(31.1%)	
10-15	33(20.5%)	38(23.6%)	
≥ 15	73(45.3%)	39(24.2%)	
***Family history of DM***			
No	50(36.8%)	53(33.1%)	0.512
Yes	86(63.2%)	107(66.9%)	
Body mass index (kg/m^2^)	26.13±5.83	28.55±4.91	<0.0001
Systolic blood pressure (mmHg)	135.51±19.7	136.88±20.45	0.567
Diastolic blood pressure (mmHg)	82.42±10.25	81.41±10.72	0.416
High density lipoprotein (mg/dl)	27.03±9.86	38.32±10.61	<0.0001
Low density lipoprotein (mg/dl)	90.94±28.94	103.24±41.94	0.087
Cholesterol (mg/dl)	166.01±59.97	172.57±49.27	0.563
Triglyceride (mg/dl)	143.58±65.76	159.45±67.57	0.252
Serum Creatinine (mg/dl)	1.33±0.66	1.19±0.68	0.240
HbA1c (%)	10.1±2.55	10.0±1.81	0.80

Data presented as mean ± standard deviation or n (%) p-value < 0.05 was considered statistically significant.

Overall, 85.67% case group (males 64.56%); females 21.11%) and 35.3% control group (males 22.9%; females 12.4%) have anemia. Mean Hb level was 10.49 g/dL in case group and 13.39g/dL in control group (p<0.0001). All other parameters of CBC were significantly different among both groups except for MCV and MCH. (Table-II)

**Table II:** Comparison of Complete Blood Count (CBC) levels between case and control groups.

Complete Blood Count (CBC)	Case	Control	P-value
N	161	161	-
Hemoglobin (g/dL)	10.49±2.07	13.39±2.06	<0.0001
***Hemoglobin deficiency***			
Males (<13 g/dL)	104(64.56%)	37(22.9%)	<0.0001
Females (<12 g/dL)	34(21.11%)	20(12.4%)	0.001
***Hemoglobin levels***			
Males (>13 g/dL)	15(9.32%)	82(50.93%)	<0.0001
Females (>12 g/dL)	8(4.97%)	22(13.66%)	<0.0001
RBC (x 10^6^/µL)	3.78±0.64	4.73±0.63	<0.0001
MCV (fl)	84.12±7.41	82.84±7.7	0.130
MCH (pg)	27.28±5.95	28.07±3.29	0.141
MCHC (g/dL)	31.81±1.8	33.65±1.95	<0.0001
TLC (x 10^3^/ul)	13.26±4.58	8.5±2.07	<0.0001
Neutrophils count (%)	79.21±9.16	68.33±8.81	<0.0001
Lymphocytes count (%)	17.67±8.84	27.45±8.78	<0.0001
Monocytes count (%)	1.5±0.65	1.95±0.64	<0.0001
Eosinophils count (%)	1.62±0.75	2.27±1.14	<0.0001
Platelets count (g10^3^/ul)	345.3±123.84	244.5±83.08	<0.0001

Data presented as mean ± standard deviation. p-value < 0.05 was considered statistically significant.

By using UT classification system people were observed in grade 0 (stage B 0.6%), in Grade-I (stage A 0.6%, stage B 3.7% and stage C1.2%) and in Grade-II (stage B 13%). While, in Grade-III (stage B 73.1%, stage C 3.1% and stage D7.5%) people were observed.

The odd ratio 95% confidence interval of clinical parameters between case and control groups is shown in Table-III. Non-significant association was observed for duration of diabetes, BMI, HDL and HbA1c between both case and control groups.

**Table III:** Odd Ratio 95% confidence interval (OR (95% CI)) of clinical parameters between case and control groups.

Parameters	Case		Control	

	OR (95% CI)	P-value	OR (95% CI)	P-value
Duration of DM	1.34(0.985-1.826)	0.062	1.10(0.782-1.54)	0.583
BMI	1.005(0.923-1.090)	0.905	1.049(0.974-1.129)	0.208
HDL	1.025(0.957-1.097)	0.487	0.983(0.92-1.051)	0.623
HbA1c	0.842(0.656-1.08)	0.176	1.006(0.731-1.385)	0.97

## DISCUSSION

In this prospective case control study, anemia was found to be a significant association in people with DFU (85.66%) compared to age and sex matched people without DFU (35.3%). Majority of cases had normocytic normochromic anemia (74.5%) followed by microcytic (24.8%) and megaloblastic (0.62%) anemia.

In our study, high frequency of anemia in people with DFU was comparable to recent studies that also showed the high prevalence of anemia 51.9%-85.3% in people with DFU.^21^,^22^ There were more males than females who presented with DFU and this was similar to earlier reports.^22^ In our study, the glycemic control (HbA1c) was poor and comparable in both case and control groups. It is well established that HbA1c levels can be affected by conditions unrelated to diabetes including anemia.^23^ Although, HbA1c was identical in both groups, HbA1c in case group may not be true indicator of glycemic control in the presence of anemia. In this study, people in both groups were obese and had low HDL levels indicating underlying insulin resistance but controls had significantly higher BMI and lower HDL levels. Regarding the duration of diabetes, there were more people with prolonged duration of diabetes i.e. ≥ 15 years in case group, but absence of anemia in control group with similar duration of diabetes highlights the fact that anemia was associated with DFU and not merely due to prolong history of the disease.

Though, the finding of anemia in people with diabetes is frequently reported in context of chronic kidney disease (CKD) but the baseline renal functions were normal in both the groups in our study.^24^,^25^ The most probable factors causing anemia in people with DFU are presence of chronic inflammation, involvement of bone leading to osteomyelitis, repeated debridement, poor dietary intake, drug induced or combination of these factors. In chronic inflammation, anaemia most probably occur in three different ways: at the iron level (insufficient levels of iron), Epo-Epo receptor level and at erythroid precursor level.^14^ Erythropoietin (Epo) allows survival and proliferation of erythroid precursor cells by generating intracellular signals resulting in the prevention of apoptosis.^26^ In anemia, due to chronic inflammation increased proinflammatory cytokines have been reported, mainly TNF-α (Tumor necrosis factor alpha), IL-1 (interleukin-1) and IL-6 (interleukin-6) that can consequently cause reduction of circulating hemoglobin.^27^

The association of anemia in people with DFU not only results in delayed healing but also leads to adverse outcomes and substantial morbidity and mortality.^28^

### Limitations of the study

Although, majority of cases had normocytic anemia, minority of the cases had microcytic anemia. These cases were not further evaluated to ascertain the cause.

## CONCLUSION

Our study concludes that anaemia is strongly associated with DFU disease. Anaemia should be considered and treated as co-morbidity, while managing patients with foot ulcers.
